# Investigation of *Catechol-O-methyltransferase (COMT)* gene Val158Met polymorphism in ovarian cancer

**DOI:** 10.4274/jtgga.galenos.2020.2020.0091

**Published:** 2021-02-24

**Authors:** İpek Yağmur Abaoğlu, Seda Güleç Yılmaz, Fatma Tuba Akdeniz, Rukset Attar, Zerrin Barut, Altay Burak Dalan, Turgay İsbir

**Affiliations:** 1 Department of Molecular Medicine, Institute of Health Sciences, Yeditepe University, İstanbul, Turkey; 2 Department of Medical Biology, Yeditepe University Faculty of Medicine, İstanbul, Turkey; 3 Department of Obstetrics and Gynecology, Yeditepe University Faculty of Medicine, İstanbul, Turkey; 4 Department of Basic Medical Science, Antalya Bilim University Faculty of Dentistry, Antalya, Turkey

**Keywords:** COMT Val158Met polymorphism, catechol estrogen, ovarian cancer

## Abstract

**Objective:**

Catechol-O-methyltransferase (COMT), the product of the *COMT* gene, detoxifies the carcinogenic catechol estrogens. The aim of the present study was to examine the relationship between *COMT Val158Met* polymorphism and the risk of ovarian cancer.

**Material and Methods:**

The study groups consist of 94 individuals as a patients group with ovarian cancer (n=47) and control group (n=47). The allele and genotype frequencies were determined according to Hardy-Weinberg equilibrium (HWE). The allele and genotype frequencies. determined according to HWE. Genetic analysis were performed by real-time-polymerase chain reaction instrument, and the statistical analysis were performed by SPSS program.

**Results:**

Although no significant relationship was obtained among groups (p=0.413) regarding *COMT* gene Val158Met polymorphism, the genotype frequencies for COMT Val158Met (rs4860) polymorphism in groups was homozygote wild type GG genotype 25.5%, heterozygote GA genotype 46.8%, homozygote mutant AA genotype 27.7%.

**Conclusion:**

This study is the first to investigate the relationship between ovarian cancer and the *Val158Met* polymorphism in the *COMT* gene in a Turkish population. No statistically significant relationship was identified among genotypes belonging to the patient and control groups although sample sizes were relatively small and the analysis should be repeated in a larger cohort.

## Introduction

Ovarian cancer is the seventh leading cause of death and is the eighth most common cancer among women. It has the highest mortality rate among all gynecological cancers. The prognosis of ovarian cancer is poor, especially when the disease is diagnosed at an advanced stage (1). There are many risk factors, which change the genetic predisposition to ovarian cancer, including consumption of alcohol, obesity, aging and family history. In addition, oxidative stress, inflammation, angiogenesis, and apoptosis may alter the progression of carcinoma. Thus, controlling these factors play a crucial role in the prevention of many cancers, including ovarian carcinoma (2).

The *Catechol-O-methyltransferase (COMT)* gene is located on chromosome 22q11.2. The *COMT* gene includes six exons, of which exon 1 and 2 are noncoding (3). *COMT* gene products are expressed in many tissues, such as bone marrow, brain, bladder, heart, kidney, liver, lung, and ovary (4). The enzyme product, COMT participates in DNA repair mechanisms (5). Many polymorphisms have been identified in the coding region of the *COMT* gene including at codon 158 (G→A), codon 72 (G→T) and codon 62 (C→T). At codon 158, valine may be replaced by methionine and this alteration in the *COMT* gene lead to a decrease in the function of the protein product (6,7).

Catechols can be of endogenous or exogenous origin (8). Some evidence indicates that reactive catechol metabolites from estrogens, the catechol estrogens, have a reactive effect which may contribute to a predisposition to cancer. The COMT enzyme Catalyzes O-methylation, which has a role in inactivation of catechol estrogens (9). COMT catalyzes the transfer of the methyl group in the coenzyme SAM to the hydroxyl group in the catechols. In the *COMT* gene, the *Val158Met* polymorphism results in a decrease in the activity of this enzyme, resulting in accumulation of carcinogenic catechol estrogens (10).

The aim of the present study was to examine the relationship of the *COMT Val158Met* polymorphism to disease by comparing polymorphism expression in patients with ovarian cancer and healthy controls.

## Material and Methods

The present study investigated COMT polymorphism frequency in 94 individuals, including patients with ovarian cancer and an equal number of healthy controls. The patients (n=47) were attending the Yeditepe University Faculty of Medicine, Department of Obstetrics and Gynecology, İstanbul, Turkey. The age-matched control group (n=47) consisted of healthy females who visited the hospital for gynecologic evaluation as part of routine check up. All procedures performed in studies involving human participants were in accordance with the ethical standards of the 1975 Declaration of Helsinki guidelines and its later amendments. The study protocol was approved by Yeditepe University Faculty of Medicine Ethics Committee (approval number: 915, date: 25.10.2018). Informed consent was obtained from all participants, prior to enrollment in the study.

### Genomic DNA isolation from blood

Blood samples of participants were taken into EDTA tubes to a volume of 5 mL. DNA isolation was performed by the iPrep DNA Extraction Robot (Invitrogen, Carlsbad, California, USA). DNA purity and concentration was determined by NanoDrop (Invitrogen, Carlsbad, California, USA).

### Genotyping analysis

The allele and genotype frequencies determined according to Hardy-Weinberg equilibrium. Analysis of *COMT* gene variants performed by using the 7500 fast-real-time-polymerase chain reaction (RT-PCR) instrument (Applied Biosystems, Foster City California, USA). *COMT Val158Met (rs4860)* gene variations determined by using TaqMan Genotyping Assay probe (Applied Biosystems, Foster City, CA, USA). COMT Val158Met (rs4860) polymorphism determined by Forward 5’-GGA GCT GGG GGC CTA CTG TG-3’ and Reverse 5’-GCC CTT TTT CCA GGT CTG ACA-3’ primers. Reaction mixture and conditions were used due to manufacturer instructions. The conditions for the RT-PCR reaction were; holding stage for 10 minutes at 95 °C and 40 repeating cycles with denaturation stage for 15 seconds at 92 °C and connecting/elongation stage for 1 minute at 60 °C.

### Statistical analysis

The data obtained from genotyping was evaluated using chi-square and Fisher’s exact tests using SPSS, version 25.0 (IBM Inc., Armonk, NY, USA). Student’s t-test was used to analyze numeric values. A p<0.05 were considered statistically significant.

## Results

As a result of the demographic data analysis, body mass index (BMI), body surface area and fasting blood glucose values in ovarian cancer patients were found to be significantly higher than the control group. There was no difference between the patients and controls regarding mean age (p=0.154). While significantly differences were deteceted in diabetes (p<0.001), and smoking (p<0.001) and these risk factors were found to be statistically meaninful among groups. Also menopausal status (premenopausal and postmenopausal), pregnancy status (number of pregnancies ≤1 or number of pregnancies >1) and parity (number of births ≤1 or number of births >1) are statistically meaningful. In the patients with ovarian cancer, the rate of postmenopausal women (80.9%) was higher than the control group (40.4%). The controls whose number of births were less than or equal to one (number of births ≤1) (54.4%) were found to be statistically higher than the patient group (26.7%). The rate of postmenopausal women (80.9%) in the patients with ovarian cancer was higher than the control group. While the rate of pregnancy status (number of pregnancies ≤1) (54.4%) in the control group was higher than the patient group. (Table 1).

In the present study, in the patients with ovarian cancer, the distribution of premenopause (80%) was analyzed to be high in comparison with postmenopause state (20%). The treatment parameters were resulted that 72.1% of the patients received adjuvant chemotherapy and 27.9% of the patients did not receive adjuvant chemotherapy. While 36.4% of the patients received neoadjuvant chemotherapy and 63.6% of the patients did not receive neoadjuvant chemotherapy. 47.5% of the patients underwent debulking surgery. Also it was observed that the rates of being pregnant more than once (77.8%) and a number of births (>1) were high (73.3%) in the patients with ovarian cancer. The ratio of patients at stage 3 (42.9%) were found to be high as compared to stage 1 (23.8%), stage 2 (23.8%) and stage 4 (14.3%). When we evaluated the tumors in terms of cell types, epithelial tumors were found at a high rate (92.3%) in comparison to the other types. Serous epithelial tumors from epithelial tumor types were calculated in a ratio of 56.4%. The ratio of sex-cord stromal tumors (5.3%) was found to be higher than germ cell tumors (2.6%).

The allele and genotype frequencies for *COMT Val158Met (rs4860)* polymorphism in groups are shown in Table 2. There was no significant difference between the proportions of patients and healthy controls in terms of polymorphism frequency (p=0.413). However, there was a tendency for patients to express the G-allele (56.4%) compared to the controls (48.9%) (p=0.389). Accordingly, there was also no difference in the expression of the A-allele between patients and controls (p=0.301).

## Discussion

Several molecular signaling pathways as hormone signaling, apoptosis, angiogenesis and oxidative stress, play crucial roles in ovarian cancer such progression of ovarian cancer (11). When the individuals with ovarian cancer are at a late-stage, 70% of patients are able to be diagnose. On the other hand, symptoms of ovarian cancer are not clear, and survival rate is almost 90% for 5-years during the first stages (12).

It is considered that COMT enzyme plays a significant role in estrogen metabolism based on many different studies (13,14,15,16). Ovarian cancer development is influenced by various risk factors such as BMI, age, tumor histology, family history of patients with ovarian cancer and smoking. Therefore, there are many factors that create a risk for ovarian cancer. In a nutshell, it is not sufficient to diagnose individuals simply according to carrying the Val158Met polymorphism in the *COMT* gene (14).

Goodman et al. (15) reported that COMT Val158Met polymorphism was not related to ovarian cancer risk due to a limited sample size. That study contained 108 cases and 106 controls from the German population. The ratio of heterozygote genotype carriers (50%) with ovarian cancer was found to be high in comparison with homozygote wild and variant type genotype carriers (25%, 25%) in the cases. There was no evidence that COMT Val158Met polymorphism increases the risk of ovarian cancer (p=0.73), and Goodman et al. (15) implied that advance studies are required to explain different combinations of polymorphisms in estrogen metabolizing enzymes.

A multigenic model constructed by eleven gene variations including the COMT Val158Met polymorphism, was performed by Delort et al. (16). Although there was no significant difference, they suggested that heterozygote COMT Val158Met polymophism was one of high-risk genotype. Heterozygote COMT Val158Met genotype could have possible effect on ovarian cancer by reducing activity of phase II enzyme that decrease the elimanition of carcinogens (16). Pan and Liao (17) investigated that COMT might be as a biomarker, which can be important factor in suppressing tumor development and treatment of cancer . In estrogen metabolism, COMT prevents DNA damage, therefore, it is called the gate-keeper (17). In light of these developments, present study will contribute to understanding molecular mechanisms for ovarian cancer.

There are two meta-analyses perfomed in order to determine the role of COMT Val158Met polymorphism in ovarian cancer susceptibility and both of them could not find any associations (8,18). But it should not be forgotten that meta-analysis data could be tested in clinical studies by recruiting homogeneous patients and controls. Moreover, in meta-analysis there are population bias caused by homogeneity, and they did not implicate any data for Turkish population. Thus, our study aim to provide reliable data about the role of COMT Val158Met polymorphism in ovarian cancer due to these uncertain results.

## Conclusion

As *COMT* polymorphisms affect enzyme capacity, the COMT gene has a significant role in estrogen metabolism and thus on hormone dependent gynecological cancers. Although, there were no statistically significant differences between the patient and control groups in the present study this may have been due to the small sample sizes. Therefore associations between *COMT* gene variations and ovarian cancer risk should be investigated in larger sample sized studies in a Turkish population.

## Figures and Tables

**Table 1:**
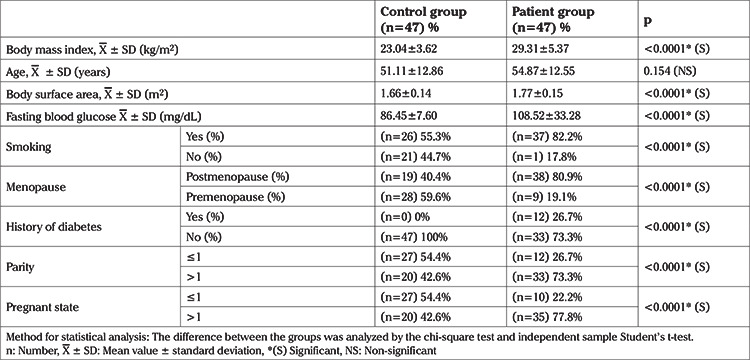
Demographic data related to the patients with ovarian cancer and healthy controls

**Table 2:**
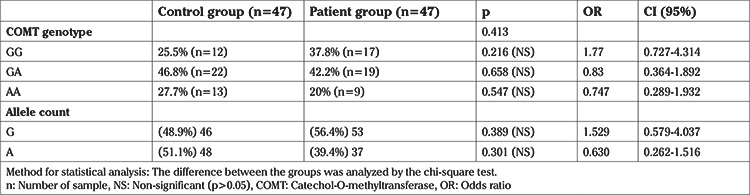
The genotype and allele distributions for the COMT gene between the patient and control groups
